# Topics of the Month

**Published:** 1895-12

**Authors:** 


### TOPICS OF THE MONTH.

It is rumored that the water bureau is entertaining a plan to place water meters in the houses of water consumers. The object, of course, is to prevent waste of water or its extravagant use. We can understand the propriety of placing safeguards wherever there is undue waste, which is likely to happen in certain factories, livery stables and public buildings. But it cannot be accentuated too strongly that such restriction of the use of water in private families would be a violation of one of the first principles of house sanitation. Theoretically, water should be as free as air, and only such a price fixed on its use as reimburses the city for its outlay in piping, pumping and carrying on the necessary expenses of the water department. If the pumping or storage capacity is inadequate it should be increased.

To enjoy the free and unrestricted use of water, after having paid the nominal fee for its conduct into the premises occupied by a householder, becomes a sanitary adjunct of great potentiality. To measure it through a meter, thereby limiting its proper use, reduces its sanitary value to a minimum. We hope the health department of Buffalo will resist such an innovation, should it be seriously proposed, with all the force of influence that it can command.

---

In another department of this issue is published the plan of organisation of the National Confederation of State Medical Examining and Licensing Boards. Dr. Charles McIntire, of Easton, Pa., is the chairman of the committee, and he invites any criticism on the report, with a view to its greater perfection before its final adoption at the next annual meeting of the confederation, May, 1896. We hope every friend of improved methods in medical education will scrutinise this report with great care and offer to Dr. McIntire any suggestions that may seem fitting and proper.

Francis Schlatter is the latest contribution of the period to the faith cure apostles. To call him a healer is a mistake. This is not a period of miracles nor is it decent for a man to claim Divine power. This is an age of cold, hard facts, and a man can only acquire sufficient knowledge to enable him to begin medical practice through long, weary years spent in study in the laboratory, hospital and medical college. Schlatter is an Alsacean peasant, with some education and intelligence, who settled on Long Island a few years ago as a shoemaker. He was always noted in his home village as a man with queer views and is reported to have had "visions." About a year ago he turned up in New Mexico, claiming to be a New Messiah with extraordinary power of curing diseases. After a reported imprisonment for falsely claiming Divine powers he reappears in Denver, where his "wonderful" cures have lately attracted attention. Threatened with incarceration, he has mysteriously departed from Denver, announcing that he will next set up his Divine cure-shop in Chicago.

It seems to be about time for intelligent people to discountenance the ridiculous performances with which this man Schlatter, and all of his kidney, are deceiving people who are ignorant or silly enough to listen to him.

---

Physicians in Buffalo are interested in improved street railway service. The Buffalo railway company has long been tried and found wanting. It is not progressive, accommodating or courteous. Its system is wholly inadequate to meet the demands of traffic, its cars are unsanitary and it subjects its motor men to undue exposure in serving cars without vestibuled ends. The Buffalo electric traction company promises something better. Let us try the new company, for nothing could be worse than the present system. Dr. Byron H. Daggett, of Franklin street, in a recent interview, (*Buffalo Express*, November 27, 1895,) said some things which deserve to be repeated :

Are you opposed to a new company? he was asked.

I want the arteries of traffic and travel to be multiplied in this city until it has all it needs, which it hasn't now, he said emphatically. I want the street cars to run past my door, one every minute, oftener if it will pay. Why, the arteries carry nourishment to the various parts of the body. Just so with transportation lines in cities.

Property here, in spite of all booms, is not worth a cent more than it was twenty-five years ago. Why ? Because it is not sought as a residence street and through lack of street car facilities business hasn't got started this way. The thing to increase the value of property here, the thing to make the property useful, is an electric railway line. It seems to be human nature to kick. People opposed the putting down of a new pavement here, but they wouldn't like to have the old stone pavement substituted for asphalt now. People who object to the construction of street car lines here now, would be the first to object to having them removed after they had begun operating.

Take any street in any city in the world which has street car service, especially electric railways, and you will be unable to find anywhere a majority of its resident and property-owners in favor of removing the lines to any other street. We need a far more extended and better service in this city. We need competition. Now, let's wake up and have it.

---

THE New Haven County Medical Association has passed a set of resolutions declaring that other states are rejecting by their examining boards graduates of colleges who in Connecticut are admitted to practise without examination; that, in consequence, that state is becoming a dumping-ground for undesirable practitioners; and that the state committee on legislation be instructed to advocate an amendment of the law so that all candidates for registration as doctors under the medical practice act be required to pass an examination, as is now the case in New York, Pennsylvania and other states.

